# Towards recycling of challenging waste fractions: Identifying flame retardants in plastics with optical spectroscopic techniques

**DOI:** 10.1177/0734242X221084053

**Published:** 2022-03-25

**Authors:** Tuomas Sormunen, Sanna Uusitalo, Hannu Lindström, Kirsi Immonen, Juha Mannila, Janne Paaso, Sari Järvinen

**Affiliations:** 1 VTT Technical Research Centre of Finland Ltd., Oulu, Finland; 2 VTT Technical Research Centre of Finland Ltd., Tampere, Finland

**Keywords:** Plastic, sorting, recycling, additives, near-infrared, Fourier-transform infrared, Raman, hyperspectral imaging

## Abstract

The use of plastics is rapidly rising around the world causing a major challenge for recycling. Lately, a lot of emphasis has been put on recycling of packaging plastics, but, in addition, there are high volume domains with low recycling rate such as automotive, building and construction, and electric and electronic equipment. Waste plastics from these domains often contain additives that restrict their recycling due to the hazardousness and challenges they bring to chemical and mechanical recycling. As such, the first step for enabling the reuse of these fractions is the identification of these additives in the waste plastics. This study compares the ability of different optical spectroscopy technologies to detect two different plastic additives, fire retardants ammonium polyphosphate and aluminium trihydrate, inside polypropylene plastic matrix. The detection techniques near-infrared (NIR), Fourier-transform infrared (FTIR) and Raman spectroscopy as well as hyperspectral imaging (HSI) in the short-wavelength infrared (SWIR) and mid-wavelength infrared (MWIR) range were evaluated. The results indicate that Raman, NIR and SWIR HSI have the potential to detect these additives inside the plastic matrix even at relatively low concentrations. As such, utilising these methods has the possibility to facilitate sorting and recycling of as of yet unused plastic waste streams, although more research is needed in applying them in actual waste sorting facilities.

## Introduction

Plastics are versatile materials used in different applications worldwide. The annual world production of plastics has increased from 1.5 million tonnes (1950) to 359 million tonnes (2018), of which 17% is produced in Europe (Plastics Europe, 2019). The problem often associated with plastic is not in its use, but its end-of-life. By 2015, about 6300 Mt of plastic waste has been generated worldwide, of which around 9% has been recycled, 12% incinerated, and 79% accumulated in landfills or the natural environment (Geyer et al., 2017). The alternatives to recycling manifest multiple problems. In landfilling, there is a major concern of chemicals leaching from waste plastics that contaminate the soil and groundwater; the decomposition of these wastes also releases large amounts of carbon dioxide (Okunola et al., 2019). In incineration, waste plastics may release hazardous chemicals as well as carbon dioxide to the atmosphere (Nagy and Kuti, 2016). Thus, increasing the rate of recycling is a necessity, along with reducing consumption and redesigning plastic products, to tackle these ecological concerns.

The packaging sector uses the most plastic: in Europe in 2018, 39% of plastic was used in this domain. Besides packaging, other areas that utilise large amounts of plastics are electrical and electronic equipment, automotive industry as well as building and construction. These three domains constitute 36% of the plastic demand in Europe, almost equalling the demand for packaging (Plastics Europe, 2019). Packaging plastics, particularly used in food-contact, are subject to high safety requirements (Kato and Conte-Junior, 2021), and thus pose low risk in recycling. However, plastics from the other three domains contain significant amounts of harmful additives that provide many challenges, but also a potential for recycling of plastic components. For example, 6% of annually produced plastics in Europe are used for electric and electronic equipment, and the collection rate of waste electric and electronic equipment (WEEE) is quite high, 47% in 2018 (Eurostat, 2021). Currently, most of the WEEE are incinerated, but if a safe and feasible recycling process could be identified, the already collected plastics fraction could be efficiently recycled into new applications, notably increasing the share of recycled plastic materials on the global scale.

The average amount of additives in plastics is roughly 7% by weight (wt%) (Geyer et al., 2017). The additives can be mainly divided into the following four categories:

1. Functional additives such as flame retardants (3–25 wt%), stabilisers, plasticisers (10–70 wt%), slip agents, foaming agents and antistatic agents.
2. Colourants including different pigments.
3. Fillers (up to 50 wt%) such as calcium carbonate, barium sulphate, mica and clay.
4. Reinforcement agents (15–30 wt%) such as different fibres.

Each of them plays a significant role in providing functional properties for the plastic product (Hahladakis et al., 2018) and is widely used in non-packaging plastics. Typically, the hazardous additives are not bound in the plastics and are able to migrate out, causing potential health and environmental risks during use and recycling. In plastic recycling, generally, it is important to avoid cross-contamination of different grades of plastics (Eriksen et al., 2018); thus, the separation of plastics according to their composition of additive concentration is a necessary step (Hahladakis et al., 2018). Efficient methods are required to sort the mixed plastic waste, but the volume of the waste stream and sorted fractions must be sufficient for recycling to be economical (Stenmarck et al., 2017). Thus, a multitude of different additives has to be identified in these challenging waste plastic streams to classify materials for further specific reprocessing, enabling both the safe recycling of plastic waste with no hazardous materials and the possibility of recycling waste containing these chemicals.

In this light, it is clear that to enable enhanced recycling of these plastic fractions, accurate identification of additives inside the polymer is required. The existing methods to solve the challenge of additive identification from plastic waste streams rely on tedious methods such as gas chromatography (Król et al., 2012), mass spectrometry (Binici et al., 2013) and scanning electron microscopy and energy-dispersive spectroscopy (SEM-EDS) (Taurino et al., 2010) that are not feasible for online use. Another solution is to use X-ray fluorescence spectrometry: it utilises electromagnetic radiation to probe the plastic for heavy elements such as bromine (as in brominated flame retardants (BFRs)) (Aldrian et al., 2015); however, this method is often costly for online use, and does not provide molecular information. Optical detection methods, however, offer several benefits including fast analysis and possibility for online detection, but the suitable technology often depends on the application and the type of additive. For example, absorbance spectroscopy and Raman spectroscopy have been shown to be effective in polymer classification, and they often complement each other as they both belong to vibrational spectroscopy group (Bart, 2006; Hummel, 2012).

This study evaluates a variety of optical measurement modalities in order to find suitable techniques to facilitate enhanced recycling of challenging plastic waste fractions. The experimental work focuses on the efficiency of Raman, near-infrared (NIR) and Fourier-transform infrared (FTIR) spectroscopy as well as short-wavelength infrared (SWIR) and mid-wavelength infrared (MWIR) hyperspectral imaging (HSI) for identification and quantification of two flame retardant additives, aluminium trihydroxide (ATH) and ammonium polyphosphate (APP) in polypropylene (PP) plastic matrix. Two different approaches of data analysis are used – the classical peak height analysis and the versatile chemometric tool partial least squares (PLS) regression.

## Related work – optical methods for additive identification

Raman spectroscopy is based on inelastic Raman scattering of photons and is a relatively weak phenomenon (Vašková, 2011). Raman belongs to vibrational spectroscopy together with infrared (IR) spectroscopy, but it has better spatial resolution with wide spectral range and narrow spectral bands giving it the benefit of distinguished fingerprint spectra with less complication due to peak overlap in mixtures compared to absorbance spectroscopy (Giles et al., 1999). The challenges with Raman are the interference coming from fluorescence, weak signal strength, and thus low signal-to-noise ratio and the possible heating of the coloured samples due to high laser power. The interference coming from fluorescence can be minimised by choosing the laser wavelength on the NIR range. Identification of different plastic types with Raman spectroscopy has been studied previously using both multi-sensor fusion techniques utilising Raman and laser-induced breakdown spectroscopy (LIBS) (Shameem et al., 2017) and Raman spectroscopy on its own (Qu et al., 2006; Tsuchida et al., 2009). Typically, the fluorescence challenge with Raman spectroscopy rises from the presence of colouring agents in plastics. In a recent publication, a combination of LIBS and Raman system had been used for the rapid identification of post-consumer plastics (Shameem et al., 2017). The plastics were categorised using statistical tools to analyse the collected spectral features of the samples. According to their research, the information from LIBS and Raman is sufficient for extensive plastic analysis as the information provided by Raman spectroscopy reached 100% discrimination level for the clear plastics, whereas the LIBS technique could distinguish the coloured samples (Shameem et al., 2017). A study analysing plastic additives evaluated the suitability of Raman for plasticisers analysis including adipate and phthalate ester contents in polyvinyl chloride (PVC) (Berg and Otero, 2006; Nørbygaard and Berg, 2004). The results showed that the absence of any Raman band in the 1020–1060 cm^−1^ wavelength range can be used to confirm that the PVC does not contain significant amounts of phthalate esters, whereas the case of adipate esters is not as straightforward due to other aliphatic compounds giving the same spectral fingerprint. To our knowledge, the detection of APP and ATH has not been previously studied using Raman spectroscopy.

Absorbance spectroscopy is commonly used for material analysis as it is a well-known technology and can often distinguish single parameters with inexpensive and compact sensors. It provides a molecule spectrum defined by the absorbance of photons at specific wavelengths, which reveal the structure of that molecule. Absorbance and Raman spectroscopy are often complementary techniques: with centrosymmetric molecules, symmetric vibrations generate either Raman scattering or IR absorbance but rarely both (Vašková, 2011). For example, ring breathing in benzene structure is active in Raman, whereas it is not active in IR (Giles et al., 1999). IR is a commonly used method in pure plastic identification. The current recycling systems utilise mainly NIR spectrometers for plastic identification, although this wavelength range has challenges with dark plastics (Inada et al., 2001). MWIR range is not disturbed by colourants, but has more sensitivity towards the surface state of the sample material (Shameem et al., 2017). However, purely MWIR-based commercial sorting units are as of yet not available. Nonetheless, MWIR spectroscopy has recently been used to identify plastics dyed with carbon black (Rozenstein et al., 2017).

FTIR spectroscopy is a commonly used technique to obtain an IR spectrum of absorption or emission of a solid, liquid or gas. An FTIR spectrometer can simultaneously collect high spectral resolution data over a wide spectral range. FTIR techniques have been widely utilised in several fields of research to analyse and characterise plastic and associated additives. For example, chemical changes of polylactic acid (PLA) and additives used in recycling processes to improve recyclability have been studied using FTIR techniques (Beltrán et al., 2019). FTIR has been used as a non-destructive measurement method in quality analysis of the most common plastic, polyethylene (PE), in bio-waste treatment facilities (Alassali et al., 2018). PE and PP degradability can also be characterised by FTIR spectroscopy (Aldas et al., 2018). Characterisation of plastic additives used in food packaging materials is often carried out using FTIR spectroscopy to ensure that regulations of stability, purity and toxicity are met (Cherif Lahimer et al., 2017; Mauricio-Iglesias et al., 2009). Albeit the method is mainly suitable for very high micro-plastic (>1%) concentration samples, the advantages are cost efficiency and relatively short time needed for the measurement (Hahn et al., 2019). Prior to this study, the attenuated total reflection (ATR)-FTIR technique has been studied for additive-containing PP sample analysis to recognise the formation of the oxidation products in the polymer after exposure to natural weathering (Barbeş et al., 2014). The PP samples contained 1%, 2% and 3% w/w synthetic antioxidant Irganox 1010 from Ciba (Basel, Austria) and anti-caking agent calcium stearate as additives. The additives in PP films were successfully characterised by ATR-FTIR. The study showed that the addition of additives in PP powders could be monitored in different environmental conditions.

HSI is a fast growing technology due to the benefits of combining spatial data with spectroscopy, generating three-dimensional (3D) data with two spatial and a spectral dimension. It is an all-around term used for sensors operating on different wavelength ranges. Like FTIR and NIR spectroscopy, HSI is based upon molecular vibrations induced by electromagnetic radiations, and the characteristic spectral features given by the absorbance of the sample. HSI has been used in pure plastic identification (Serranti et al., 2011; Ulrici et al., 2013). However, for detection of plastic additives, only a handful of studies have been conducted (Amigo et al., 2015; Bonifazi et al., 2021; Caballero et al., 2019). The studies are related to identifying BFRs in plastics, and the used wavelength ranges were in the SWIR range in all studies. In this study, we explore the aforementioned optical spectroscopy techniques for the detection of fire retardants APP and ATH.

## Materials and methods

This study examines different identification methods for flame retardants ATH and APP contained in PP. These additives raise interest as they are widely applied in plastic products. ATH is the largest volume metal hydrate type flame retardant added in thermoplastics and thermosetting materials in high amounts (even up to 60%) to give proper flame retarding effect through the release of chemically bonded water. APP, however, is a common char former type flame retardant often found in levels of 20%–30% in plastics (Innes and Innes, 2004). The present analysis compares two different data analysis approaches for additive detection based on spectra obtained from different modalities: peak height calibration and PLS.

### Fabrication of PP plastic samples with APP and ATH additives

Planar samples of shape of universal test specimens (‘dog-bones’) with varying load of flame retardants were prepared by melt-compounding of additives into thermoplastic matrix followed by timely injection moulding, using DSM Xplore 15cc Microcompounder (Sittard, The Netherlands) and Thermo Haake Minijet Injection moulding machine (Karslruhe, Germany). PP BE170MO from Borealis (Vienna, Austria) was used as matrix polymer and ATH (FordaGard M6B from LKAB Minerals, Luleå, Sweden) and APP (Exolit AP 750 from Clariant, Muttenz, Switzerland), respectively, as flame retardants. Both additives were loaded in amounts of 1, 2, 4, 8 and 16 wt% in PP, and plain reference PP samples without additives were prepared, respectively.

### Raman measurements

The Raman measurements were executed using a traditional continuous-wave (CW) excitation RamanRxn2™ Hybrid Analyser by Kaiser Optical Systems (Ann Arbor, Michigan, United States). The CW Raman had 785 nm excitation wavelength with spectral coverage of 150–1875 cm^−1^. To collect the Raman spectra from the samples, non-contact MR Probe from Kaiser Optical Systems was used in connection to a sample chamber. The samples were manually shifted to collect 10 measurement points from the sample surface with exposure time of 10 seconds.

### NIR spectroscopy

Cary 5000 VIS-IR spectrophotometer by Agilent (Santa Clara, California, United States) was utilised to measure NIR spectrum of the samples in 1000–2000 nm wavelength range. The visible (VIS) range was not included, as the colour features might correlate with additive concentration without necessarily containing relevant features. The samples were measured with two measurement geometries: integrating sphere accessory DRA-2500 or Universal Measurement Accessory (UMA). Reflectance measurements for the APP-doped samples were performed using UMA, in which case reflection in 45° angle relative to surface normal was measured. Reflectance measurements for ATH-doped samples were done using integrating sphere in diffuse reflectance mode, which omits specular reflected component. Highly Lambertian Labsphere Spectralon SRS-99 diffuse reflectance standard was used as a white reference.

### FTIR measurements

FTIR spectroscopy measurements of the samples were done using Diffuse Reflectance Infrared Fourier-Transform Spectroscopy (DRIFTS). DRIFTS accessory measures only diffuse reflection and omits specular reflected component. The reflectance reference spectrum was measured from diffuse aluminium surface. Each measured spectrum consists of 100 scans and spectral data were collected in 1–10 µm wavelength range.

### HSI

Two commercial HSI cameras by Specim were used for imaging the samples. The Specim SWIR camera operates in the wavelength range 970–2530 nm, on which range the PP + ATH set was imaged. The PP + APP set was imaged with a sensor yielding a slightly narrower range, that is, 1000–2518 nm, due to availability issues. The other camera, Specim FX50, operates in the MWIR range 2700–5300 nm. Both sets were imaged with this range.

The cameras are integrated with the push broom scanner; the row detector scans passively a line on top of a conveyor belt, which actuates the gathering of reflected light in the y-direction of the sample. For both cameras, constant illumination with a continuous spectrum is directed at 45° angle towards the belt. Dark and white reference samples were gathered individually for each imaging, which are then used to convert the raw signal to reflectance values.

### Data analysis

Two different analysis approaches were used to evaluate the measurement modalities in two respects. The first, peak height analysis, provides information on whether or not the feature of the characteristic peak of each additive can be used to reliably predict the concentration of the additive from the polymer-additive blend spectra. The second, PLS, provides a more general framework for automatic analysis without manually correcting the spectra and selecting the features of interest; the approach can also be utilised for multivariate analysis, that is, considering more than one single additive at a time, as well as for the cases where more than one characteristic peak is available for the target additive. The use of PLS or other chemometric or machine learning approach is more suitable for online analysis, while peak height analysis is analytically more robust particularly in the case of low number of samples.

Assuming a homogeneous distribution of additive material in the samples and linear spectral mixing, the concentration of each component (PP and additive) should manifest themselves in the spectra in a linear manner. In this paradigm, if the sample contains substances A and B, the total spectrum would be a linear combination of each individual spectrum, with the weights corresponding to the concentration of each substance. This is known as the linear mixing model. Since PLS is nothing but a coordinate transform for the spectra, the linear mixing model works for transformed spectra as well: the PLS predictions should correlate with the concentrations of substances A and B.

### Peak height analysis

Peak height analysis assumes that the concentration of the material components, such as the plastic additives, is proportional to the height or area of their characteristic peaks in the recorded spectrum. The quantification of the component impact through signal peak height is a straightforward process including baseline correction, spectrum normalisation, characteristic peak identification and averaging the area of that peak. In this case, it is possible to do quantification without internal peak reference as the data sets are relatively unaffected by changes in variation of analyte peak shape or position, intrinsic fluorescence or self-absorption of the signal (Giles et al., 1999). Thus, the plastic additive quantification relies directly on the information provided by the characteristic peaks.

The quantification process starts with correction of spectrum baseline by polynomial fitting to specified spectral regions. Next, the process continues with normalisation of spectra by dividing the spectrum by the mean value of the normalisation region. The following phase averages the area of the characteristic peaks and creates a linear fit to the calculated signals versus the sample concentration data. For this linear fit, we can calculate the error of prediction using the 10 individually measured sample points and comparing their result to the average result.

### PLS analysis

In previous studies, PLS has been found to be an effective tool in analysing HSI (Amigo et al., 2015; Caballero et al., 2019; Karaca et al., 2013; Ulrici et al., 2013), Raman (Allen et al., 1999; Da Silva and Wiebeck, 2019; Zhao et al., 2021) and FTIR data (Da Silva and Wiebeck, 2017; Lao et al., 2016) in the plastic domain. Previously, this method was validated in-house for the use of classifying bio-composites, that is, polymers mixed with cellulose pulp material (Sormunen et al., 2019). As such, it has been demonstrated to be applicable for mix materials, and thus could potentially be used for additives as well.

PLS is a statistical regression method that projects the dependent as well as the independent variables to a new space, which maximises the intra-specimen variance as well as the inter-specimen covariance. PLS bears similarities to principal coefficient analysis (PCA) in that it performs a transformation, for the target variable, such that a set of orthogonal coordinates in a new space is found. PLS can be thought of a PCA for both variables. PLS is applicable to multi-collinear data, such as is the case with spectral data; the reflectance values on adjacent wavelengths are highly correlated.

The representative spectra for all measurement methods were gathered by averaging over all measurements of a sample. With point-measurement techniques, several measurements were taken from one location of the sample. For HSI, pixels portraying the sample were selected by hand, and an average was taken over the whole spatial dimension.

Before pre-processing, the reflectance values of VIS-IR, SWIR, MWIR and FTIR were converted to absorbance values, since these are directly correlated with chemical component concentration according to the Beer–Lambert law. Then, the process was as follows: (1) spectral de-noising by Savitzky–Golay filter; (2) baseline removal using Zhang’s algorithm (Zhang et al., 2010); (3) standard-normal variate. Using the leave-one-out method of calibration, the concentration of the left-out sample is predicted according to the PLS regressor trained with the other samples using the maximum number of latent variables (i.e. five).

## Results and discussion

### Peak height analysis

**Figure 1.**
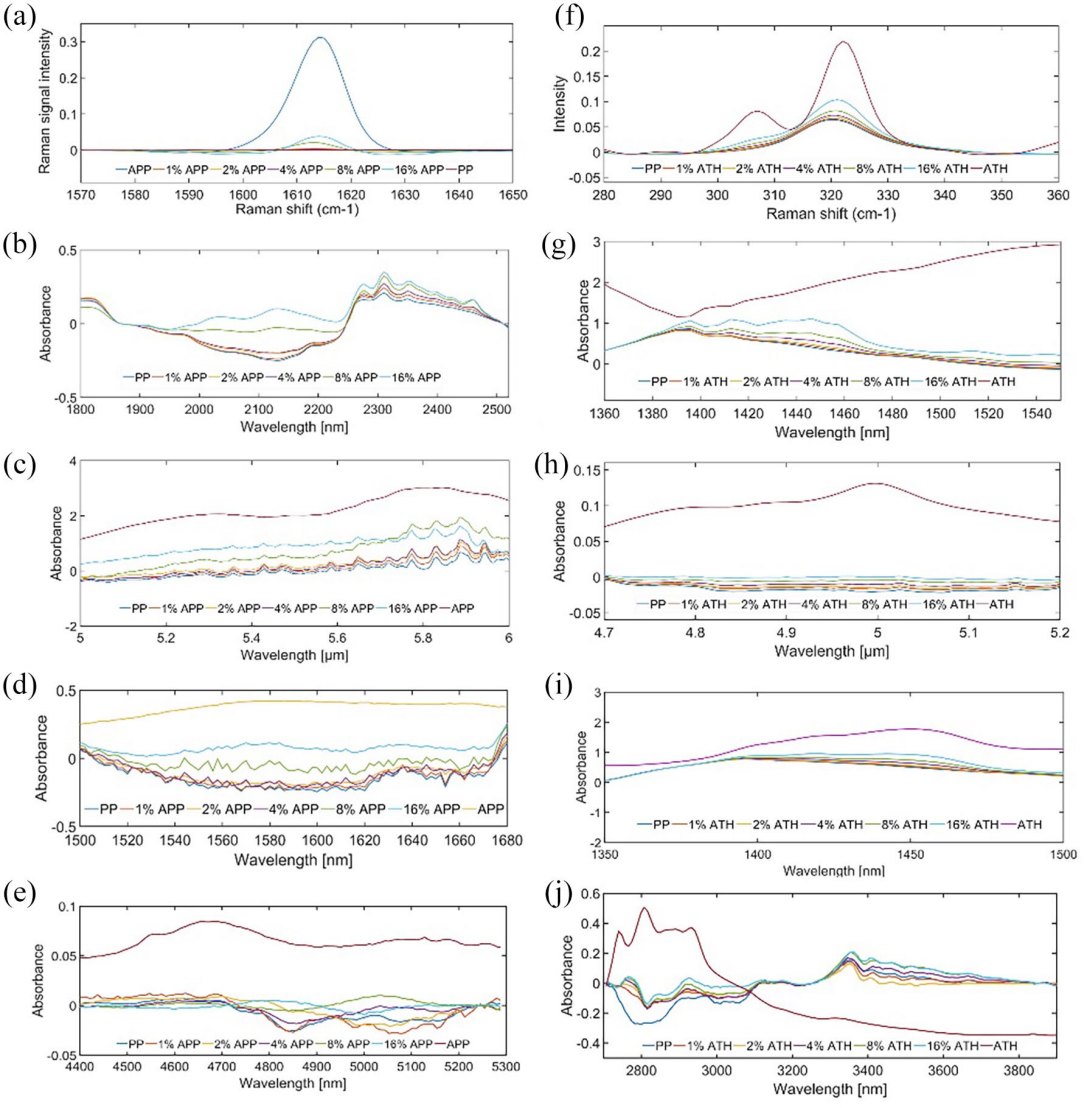
APP and ATH centred characteristic peaks with different modalities: (a) APP at 1614 cm^−1^ with 785 nm Raman, (b) APP at 2130 nm with SWIR HSI, (c) APP at 5.3 µm FTIR, (d) APP at 1.58 µm with VIS-IR, (e) APP at 4.6 µm with MWIR HSI, (f) ATH at 320 cm^−1^ recorded with Raman, (g) ATH at 1440 nm with SWIR HSI, (h) ATH at 5.0 µm recorded with FTIR, (i) ATH at 1.44 µm with VIS-IR and (j) ATH at 2.9 µm with MWIR HSI.

The peak height analysis evaluated the linear relation between APP and ATH concentration in PP through their characteristic peaks shown in Figure 1. The peak locations follow the signal shown by APP and ATH reference spectrum in the range where the PP matrix signal is in contrast low. This result shows how the APP substance generates an optical signal in PP detectable using NIR to IR absorbance as well as Raman spectroscopy. There is clear relation between the strength of the signal and the amount of additive in the PP matrix. Exception is the MWIR range for APP, where the relation is negative and clearly not caused by the additional absorption of APP. Unfortunately in this case, the best absorption area of APP coincides with PP absorption and clear relation cannot be distinguished. Therefore, the relation shown by peak height analysis is based on other correlation factors and the result is not reliable.

**Figure 2.**
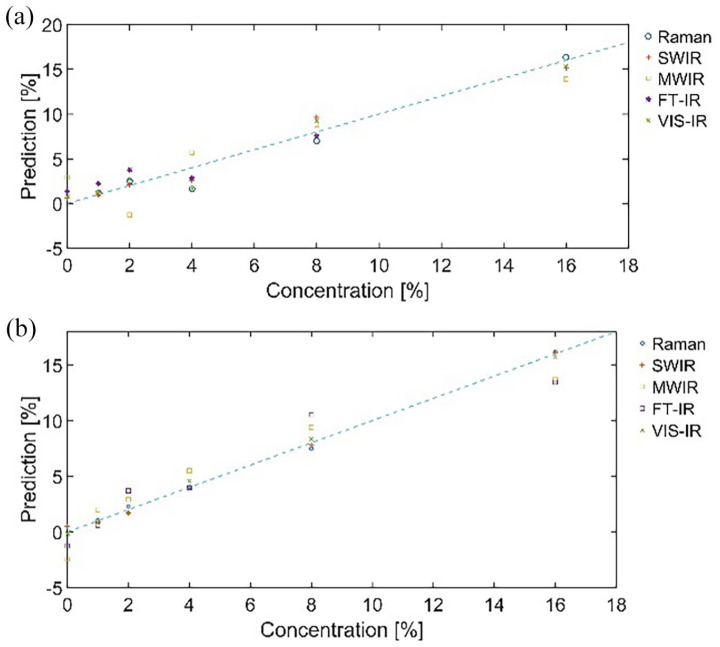
(a) APP predictions using peak height calibration calculated from Raman, SWIR HS, FTIR and VIS-IR spectral data and (b) ATH predictions using peak height calibration calculated from Raman, SWIR HS, FTIR and VIS-IR spectral data.

The signal information from the characteristic peaks can be used to create a linear prediction for the relation between signal strength and additive concentration as described in the previous section. Figure 2 presents the average predicted concentrations in relation to the reference values for Raman, SWIR HSI, FTIR, VIS-IR and MWIR data. The accuracy of these predictions is evaluated through prediction errors – Root Mean Square Error of Calibration (RMSEC) and Standard Error of Calibration (SEC).

The RMSEC and SEC for the predictions shown in Figure 2 are presented in Table 1. The results indicate that the used FTIR analysis process was not suitable for APP and ATH additive determination as FTIR shows the largest errors for the calibration. This could be related to the very shiny exterior of the plastic samples and the used Diffuse Reflectance measurement protocol. It might be possible to get better FTIR results using Attenuated Total Reflection probes. However, this additive analysis evaluates methods suitable for online analysis in future, and thus, further studies using ATR would not offer benefits for the final goal. Otherwise, VIS-IR and Raman spectroscopy give very comparable results for the calibration error. Taking into account the end goal, this gives a promising inclination for the suitability of SWIR HSI as an online tool for APP and ATH plastic additive analyses. The ability of two-dimensional (2D) imaging for averaging larger sample coverage makes the imaging also a faster and more practical tool compared to point-by-point-based Raman and VIS-IR analyses, as the signal-to-noise ratio can be effectively reduced. Moreover, it allows for distinguishing chemical differences in different parts of the plastic body material.

**Table 1.** The error of first-order fit of characteristic peak height as a function of additive concentration for each measurement modality in the case of APP and ATH.

Error (%)	Raman	SWIR	FTIR	VIS-IR	MWIR
APP, RMSEC	1.19	0.99	1.58	0.79	2.12
APP, SEC	1.22	1.08	1.71	0.87	2.32
ATH, RMSEC	0.28	0.24	1.36	0.34	1.70
ATH, SEC	0.31	0.26	1.49	0.37	1.86

SWIR: short-wavelength infrared; FTIR: Fourier-transform infrared; VIS-IR: visible infrared; MWIR: mid-wavelength infrared; APP: ammonium polyphosphate; RMSEC: Root Mean Square Error of Calibration; SEC: Standard Error of Calibration; ATH: aluminium trihydroxide.

### PLS results

**Figure 3.**
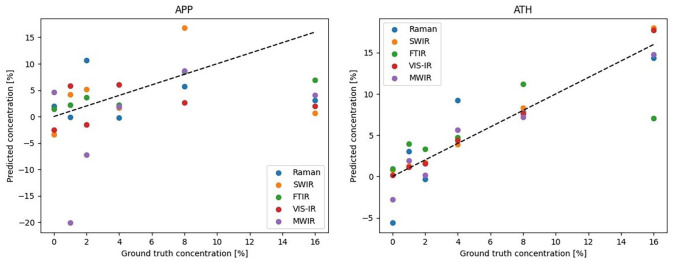
Predicted concentration values with PLS of each measurement modality for APP (left) and ATH (right). Perfect correlation is shown with the black dashed line as a reference.

The PLS calibration plots for both APP and ATH are shown in Figure 3. As previously for peak height calibration, prediction accuracy is evaluated by RMSEC and SEC for both cases; these are shown in Table 2.

**Table 2.** The error of PLS as a function of additive concentration for each measurement modality in the case of APP and ATH.

Error (%)	Raman	SWIR	FTIR	VIS-IR	MWIR
APP, RMSEC	6.71	7.63	3.92	6.73	10.80
APP, SEC	7.36	8.36	4.29	7.37	11.82
ATH, RMSEC	3.45	0.90	4.14	0.77	1.68
ATH, SEC	3.78	0.98	4.54	0.84	1.84

SWIR: short-wavelength infrared; FTIR: Fourier transform infrared; VIS-IR: visible infrared; MWIR: mid-wavelength infrared; APP: ammonium polyphosphate; RMSEC: Root Mean Square Error of Calibration; SEC: Standard Error of Calibration; ATH: aluminium trihydroxide.

As is evident for the results for APP, the error values are very high, and the calibration plots for all modalities show large deviations from linearity. It seems there is not enough data for the PLS model to capture the variation in terms of the characteristic APP peak. Indeed, as the number of samples is rather low, only five latent variables can be used in building the model, which may be an insufficient number.

For ATH, however, most of the modalities show good linear fit; the errors for SWIR and VIS-IR are below 1% and for MWIR below 2%. For Raman and FTIR, there is some discrepancy, as can be seen from the plot. This may be due to both covering a rather broad wavelength range with many features, causing the model to be unable to capture the variation caused by the additive. The utilised VIS-IR spectroscopy setup is able to capture wavelengths with a step size of 2 nm, while the SWIR HSI camera provides wavebands with step sizes of around 5.7 nm. As such, the former is able to capture more minute variations in the spectra, leading to lower errors.

In order to build a more robust chemometric model, much more data are needed, as is evident from the great difference between the peak height calibration and the PLS results. The former clearly shows that the additive peak height information can be used for accurate prediction, while the latter fails in the case of APP. As such, the model should be complemented with greater number of samples with different concentrations of additives in order to leverage more latent variables. Moreover, the model could also benefit from the use of non-averaged data; instead of using a single spectrum, the model could be calibrated using multiple spectra from the same sample.

## Conclusion

In this work, multiple optical measurement techniques based upon spectroscopy were evaluated in terms of their capability to detect additives inside plastics. Different concentrations of APP and ATH moulded in PP matrix could be accurately determined using the peak height calibration method for data obtained from Raman, NIR and SWIR HSI. The cross-validation errors in each case is low; however, for ATH, the spectral fingerprint seems to be more readily distinguishable, as evidenced by much lower RMSEC and SEC for each of these modalities. The characteristic peak of APP seems to overlap with PP, making the distinction of signal difficult.

The results indicate that the ability of HSI to detect changes in large areas of the plastic samples gives the technology good overall analysis capabilities for the additive amounts in the samples. Although the data analysis shows that the other technologies do reach similar identification and sensitivity levels, they are limited to pointwise measurement. Especially for sorting facilities, HSI technology is a versatile choice as it has the ability of scanning the width of the conveyor belt at once, allowing for using spatial in addition to spectral features. Moreover, it also allows for averaging over multiple points, leading to significantly lower noise levels.

The utilised PLS model worked for some measurement modalities in the case of ATH, but for APP, it failed to produce good results. As mentioned above, the peak height analysis results for ATH were better, implying that its spectral fingerprint is distinguishable. Nonetheless, it is clear that the model should be complemented with more samples of different concentration, and instead of an averaged spectrum per concentration, multiple spectra from the same sample could be used. This would also open the door for utilising more modern data analysis techniques based on machine learning such as support vector machines or decision trees, particularly in the case of HSI, where the amount of collected data is huge.

Utilising the results of this study, the separation of additive-containing fraction from non-additive-containing waste in sorting facilities could potentially be achieved. This would enable harvesting plastics of low additive concentration from mostly unused fractions such as WEEE for mechanical reprocessing, while the plastics of high additive concentration can either be used for incineration or reprocessed using novel chemical recycling technologies in the future. However, much more research is needed with real-life waste plastic samples and also with different polymer types to identify the exact cases where the proposed methodologies provide accurate results, such as plastics covered with grime or dirt resulting from the collection phase. Moreover, the analytical limit of the different techniques should be established, and compared to the legislation pertaining to the highest permitted threshold of harmful additives in the output recycled plastic. Nonetheless, these results indicate that the detection of additives inside plastics using spectroscopic means is possible, providing guidance for the next steps of research this domain.

## References

[bibr1-0734242X221084053] Alassali A Moon H Picuno C , et al. (2018) Assessment of polyethylene degradation after aging through anaerobic digestion and composting. Polymer Degradation and Stability 158: 14–25.

[bibr2-0734242X221084053] Aldas M Paladines A Valle V , et al. (2018) Effect of the prodegradant-additive plastics incorporated on the polyethylene recycling. International Journal of Polymer Science 2018: 2474176.

[bibr3-0734242X221084053] Aldrian A Ledersteger A Pomberger R (2015) Monitoring of WEEE plastics in regards to brominated flame retardants using handheld XRF. Waste Management 36: 297–304. 25464945 10.1016/j.wasman.2014.10.025

[bibr4-0734242X221084053] Allen V Kalivas JH Rodrigues RG (1999) Post-consumer plastic identification using Raman spectroscopy. Applied Spectroscopy 53: 672–681.

[bibr5-0734242X221084053] Amigo JM Babamoradi H Elcoroaristizabal S (2015) Hyperspectral image analysis. A tutorial. Analytica Chimica Acta 896: 34–51. 26481986 10.1016/j.aca.2015.09.030

[bibr6-0734242X221084053] Barbeş L Rădulescu C Stihi C (2014) ATR-FTIR spectrometry characterisation of polymeric materials. Romanian Reports in Physics 66: 765–777.

[bibr7-0734242X221084053] Bart JCJ (2006) Plastics Additives: Advanced Industrial Analysis. Washington, DC: IOS Press.

[bibr8-0734242X221084053] Beltrán FR Infante C de la Orden MU , et al. (2019) Mechanical recycling of poly(lactic acid): Evaluation of a chain extender and a peroxide as additives for upgrading the recycled plastic. Journal of Cleaner Production 219: 46–56.

[bibr9-0734242X221084053] Berg RW Otero AD (2006) Analysis of adipate ester contents in poly (vinyl chloride) plastics by means of FT-Raman spectroscopy. Vibrational Spectroscopy 42: 222–225.

[bibr10-0734242X221084053] Binici B Bilsel M Karakas M , et al. (2013) An efficient GC-IDMS method for determination of PBDEs and PBB in plastic materials. Talanta 116: 417–426. 24148424 10.1016/j.talanta.2013.05.076

[bibr11-0734242X221084053] Bonifazi G Fiore L Gasbarrone R , et al. (2021) Detection of brominated plastics from E-waste by short-wave infrared spectroscopy. Recycling 6: 54.

[bibr12-0734242X221084053] Caballero D Bevilacqua M Amigo JM (2019) Application of hyperspectral imaging and chemometrics for classifying plastics with brominated flame retardants. Journal of Spectral Imaging 8: 1–16.

[bibr13-0734242X221084053] Cherif Lahimer M Ayed N Horriche J , et al. (2017) Characterization of plastic packaging additives: Food contact, stability and toxicity. Arabian Journal of Chemistry 10: S1938–S1954.

[bibr14-0734242X221084053] Da Silva DJ Wiebeck H (2017) Using PLS, iPLS and siPLS linear regressions to determine the composition of LDPE/HDPE blends: A comparison between confocal Raman and ATR-FTIR spectroscopies. Vibrational Spectroscopy 92: 259–266.

[bibr15-0734242X221084053] Da Silva DJ Wiebeck H (2019) Predicting LDPE/HDPE blend composition by CARS-PLS regression and confocal Raman spectroscopy. Polimeros 29: 1–7.

[bibr16-0734242X221084053] Eriksen MK Pivnenko K Olsson ME , et al. (2018) Contamination in plastic recycling: Influence of metals on the quality of reprocessed plastic. Waste Management 79: 595–606. 30343792 10.1016/j.wasman.2018.08.007

[bibr17-0734242X221084053] Eurostat (2021) Waste statistics – Electrical and electronic equipment. Available at: https://ec.europa.eu/eurostat/statistics-explained/index.php?title=Waste_statistics_-_electrical_and_electronic_equipment#Main_statistical_f (accessed 16 November 2021).

[bibr18-0734242X221084053] Geyer R Jambeck JR Law KL (2017) Production, use, and fate of all plastics ever made. Science Advances 3: 25–29. 10.1126/sciadv.1700782 PMC5517107 28776036

[bibr19-0734242X221084053] Giles JH Gilmore DA Denton MB (1999) Quantitative analysis using Raman spectroscopy without spectral standardization. Journal of Raman Spectroscopy 30: 767–771.

[bibr20-0734242X221084053] Hahladakis JN Velis CA Weber R , et al. (2018) An overview of chemical additives present in plastics: Migration, release, fate and environmental impact during their use, disposal and recycling. Journal of Hazardous Materials 344: 179–199. 29035713 10.1016/j.jhazmat.2017.10.014

[bibr21-0734242X221084053] Hahn A Gerdts G Völker C , et al. (2019) Using FTIRS as pre-screening method for detection of microplastic in bulk sediment samples. Science of the Total Environment 689: 341–346. 31277002 10.1016/j.scitotenv.2019.06.227

[bibr22-0734242X221084053] Hummel DO (2012) Atlas of Plastics Additives: Analysis by Spectrometric Methods. Berlin: Springer Science & Business Media.

[bibr23-0734242X221084053] Inada K Matsuda R Fujiwara C , et al. (2001) Identification of plastics by infrared absorption using InGaAsP laser diode. Resources, Conservation and Recycling 33: 131–146.

[bibr24-0734242X221084053] Innes J Innes A (2004) Plastic flame retardants: Technology and current developments. Polimeri: Časopis Za Plastiku i Gumu 25: 60-60.

[bibr25-0734242X221084053] Karaca AC Erturk A Gullu MK , et al. (2013) Automatic waste sorting using shortwave infrared hyperspectral imaging system. In: Workshop on hyperspectral image and signal processing, evolution in remote sensing, Gainesville, FL, 26–28 June. Available at: 10.1109/WHISPERS.2013.8080744

[bibr26-0734242X221084053] Kato LS Conte-Junior CA (2021) Safety of plastic food packaging : The challenges about identification and risk assessment. Polymers 13: 2077. 34202594 10.3390/polym13132077 PMC8271870

[bibr27-0734242X221084053] Król S Zabiegała B Namieśnik J (2012) Determination of polybrominated diphenyl ethers in house dust using standard addition method and gas chromatography with electron capture and mass spectrometric detection. Journal of Chromatography A 1249: 201–214. 22749362 10.1016/j.chroma.2012.06.001

[bibr28-0734242X221084053] Lao W He Y Li G , et al. (2016) The use of FTIR coupled with partial least square for quantitative analysis of the main composition of bamboo/polypropylene composites. Spectroscopy and Spectral Analysis 36: 55–59. 27228740

[bibr29-0734242X221084053] Mauricio-Iglesias M Guillard V Gontard N , et al. (2009) Application of FTIR and Raman microspectroscopy to the study of food/packaging interactions. Food Additives and Contaminants – Part A Chemistry, Analysis, Control, Exposure and Risk Assessment 26: 1515–1523. 10.1080/02652030903148306 19711216

[bibr30-0734242X221084053] Nørbygaard T Berg RW (2004) Analysis of phthalate ester content in poly (vinyl chloride) plastics by means of Fourier transform Raman spectroscopy. Applied Spectroscopy 58: 410–413. 15104810 10.1366/000370204773580248

[bibr31-0734242X221084053] Nagy Á Kuti R (2016) The environmental impact of plastic waste incineration. AARMS – Academic and Applied Research in Military and Public Management Science 15: 231–237.

[bibr32-0734242X221084053] Okunola AA Kehinde IO Oluwaseun A , et al. (2019) Public and environmental health effects of plastic wastes disposal: A review. Journal of Toxicology and Risk Assessment 5. Available at: 10.23937/2572-4061.1510021

[bibr33-0734242X221084053] Plastics Europe (2019) Plastics – The Facts 2019. An Analysis of European Plastics Production, Demand and Waste Data. Available at: https://www.plasticseurope.org/en/resources/market-data (accessed 23 September 2021).

[bibr34-0734242X221084053] Qu X Williams JS Grant ER (2006) Viable plastics recycling from end-of-life electronics. IEEE Transactions on Electronics Packaging Manufacturing 29: 25–31.

[bibr35-0734242X221084053] Rozenstein O Puckrin E Adamowski J (2017) Development of a new approach based on midwave infrared spectroscopy for post-consumer black plastic waste sorting in the recycling industry. Waste Management 68: 38–44. 28736049 10.1016/j.wasman.2017.07.023

[bibr36-0734242X221084053] Serranti S Gargiulo A Bonifazi G (2011) Characterization of post-consumer polyolefin wastes by hyperspectral imaging for quality control in recycling processes. Waste Management 31: 2217–2227. 21745732 10.1016/j.wasman.2011.06.007

[bibr37-0734242X221084053] Shameem KMM Choudhari KS Bankapur A , et al. (2017) A hybrid LIBS–Raman system combined with chemometrics: An efficient tool for plastic identification and sorting. Analytical and Bioanalytical Chemistry 409: 3299–3308. 28321503 10.1007/s00216-017-0268-z

[bibr38-0734242X221084053] Sormunen T Järvinen S Lämsä A , et al. (2019) Material sorting using hyperspectral imaging for biocomposite recycling. In: Fanguiero R (ed.) 4th International Conference on Natural Fibers – Smart Sustainable Solutions, ICNF 2019, pp.250–251. Available at: https://cris.vtt.fi/ws/portalfiles/portal/55598810/ICNF2019_sormunen_split_pdf.pdf

[bibr39-0734242X221084053] Stenmarck Å Belleza EL Fråne A , et al. (2017) Hazardous substances in plastics – Ways to increase recycling. In: Nordic Council of Ministers. Available at: 10.6027/TN2017-505

[bibr40-0734242X221084053] Taurino R Pozzi P Zanasi T (2010) Facile characterization of polymer fractions from waste electrical and electronic equipment (WEEE) for mechanical recycling. Waste Management 30: 2601–2607. 20843675 10.1016/j.wasman.2010.07.014

[bibr41-0734242X221084053] Tsuchida A Kawazumi H Kazuyoshi A , et al. (2009) Identification of shredded plastics in milliseconds using Raman spectroscopy for recycling. In: Proceedings of IEEE Sensors, Christchurch, New Zealand, 25–28 October, pp.1473–1476. Available at: 10.1109/ICSENS.2009.5398454

[bibr42-0734242X221084053] Ulrici A Serranti S Ferrari C , et al. (2013) Efficient chemometric strategies for PET-PLA discrimination in recycling plants using hyperspectral imaging. Chemometrics and Intelligent Laboratory Systems 122: 31–39.

[bibr43-0734242X221084053] Vašková H (2011) A powerful tool for material identification: Raman spectroscopy. International Journal of Mathematical Models and Methods in Applied Sciences 5: 1205–1212.

[bibr44-0734242X221084053] Zhang ZM Chen S Liang YZ (2010) Baseline correction using adaptive iteratively reweighted penalized least squares. Analyst 135: 1138–1146. 20419267 10.1039/b922045c

[bibr45-0734242X221084053] Zhao Y Lin J Liu J , et al. (2021) Study on the method of identifying waste plastic materials based on Raman spectroscopy. Spectroscopy and Spectral Analysis 41: 122–126.

